# Supplementary material for: Activation of Adenosine Triphosphate-Gated Purinergic 2 Receptor Channels by Transient Receptor Potential Vanilloid Subtype 4 in Cough Hypersensitivity
Source: Biomolecules. 2025 Feb 14;15(2):285. doi: 10.3390/biom15020285 (PMC11852612; doi:10.3390/biom15020285)
Supplement: Supplementary file 1 [file biomolecules-15-00285-s001.zip › biomolecules-3352736-supplementary.pdf]

Supplementary legends

Supplementary Table 1. Changes in I_{ATP} and relative inhibition rate after different drug interventions in healthy control group and chronic cough model group

Supplementary Table 2. Changes in I_{ATP} and relative inhibition rate after different drug interventions in chronic cough model group

Supplementary Table S1. Changes in I_{ATP} and relative inhibition rate after different drug interventions in healthy control group and chronic cough model group

Groups	I_{ATP} (pA)	vs Healthy control group	vs Chronic cough model group
		inhibition rate（%）	
Healthy control group	297.75 ± 32.15	/	/
Chronic cough model group	451.00 ± 33.72	153.24 ± 18.11%	/
Control+A317491 group	124.25 ± 16.45	41.46 ± 1.35%	
Control+PSB12062 group	162.25 ± 5.56	56.54 ± 5.77%	/
Control+A804598 group	146.00 ± 2.94	49.95 ± 5.03%	/
Model+A317491 group	277.50 ± 26.38	/	61.65 ± 3.68%
Model+PSB12062 group	263.75 ± 13.67	/	59.32 ± 6.97%
Model+A804598 group	236.75 ± 20.54	/	51.03 ± 6.94%

Supplementary Table S2. Changes in I_{ATP} and relative inhibition rate after different drug interventions in chronic cough model group

Groups	I_{ATP} (pA)	vs Chronic cough model group	vs GSK1016790A group
		inhibition rate（%）	
GSK1016790A group	626.00 ± 52.37	154.73 ± 29.39%	/
HC067047 group	230.50 ± 19.80	61.86 ± 7.15%	/
GSK1016790A+A317491 group	153.25 ± 8.65	40.26 ± 4.88%	26.26 ± 2.07%
GSK1016790A+PSB12062 group	212.75 ± 27.80	56.00 ± 1.87%	36.93 ± 5.91%

GSK1016790A+A804598 group	210.50 ± 9.98	56.07 ± 9.06%	36.46 ± 2.62%/
GSK1016790A+A317491/PSB12062/A804598 group	83.00±14.76	19.96 ± 0.70%	13.16± 2.13%

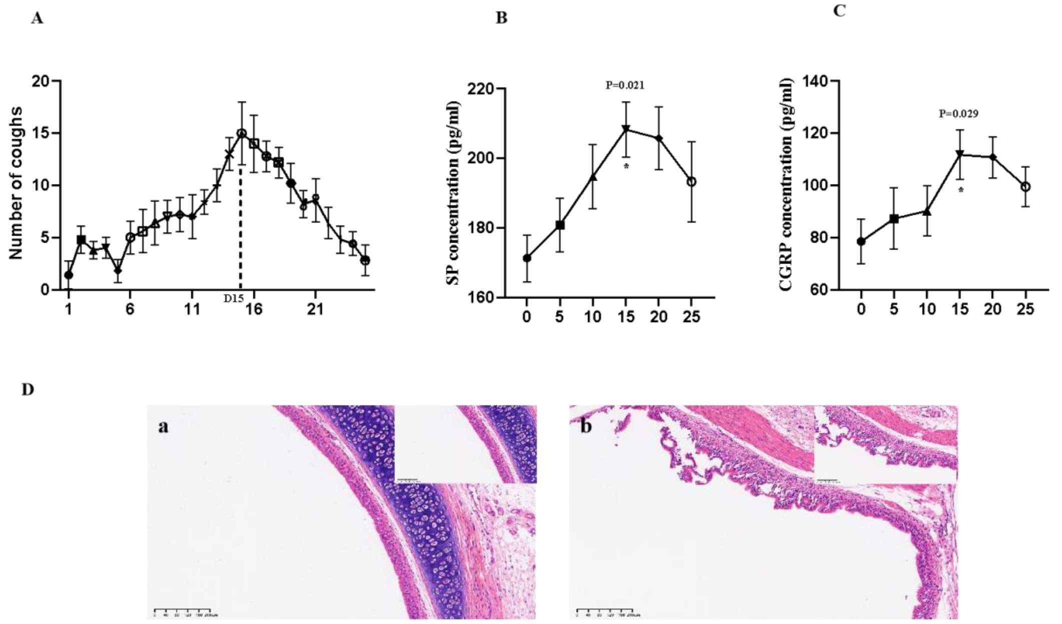

Supplemantary Figure S1. The establishment of Chronic cough guinea pig model

A: Cough responsiveness of guinea pigs inhaling 0.4M citric acid; B-C: Changes of substance P and CGRP in BALF of chronic cough guinea pigs. D: Morphological changes of the tracheal carina((a) Healthy control group; b) Chronic cough model group
